# Efficiency of Ag_3_PO_4_/TiO_2_ as a heterogeneous catalyst under solar and visible light for humic acid removal from aqueous solution

**DOI:** 10.1016/j.heliyon.2023.e15678

**Published:** 2023-04-22

**Authors:** Roya Morovati, Saeed Rajabi, Mohammad Taghi Ghaneian, Mansooreh Dehghani

**Affiliations:** a Department of Environmental Health Engineering, School of Health, Shiraz University of Medical Sciences, Shiraz, Iran; b Student Research Committee, School of Health, Shiraz University of Medical Sciences, Shiraz, Iran; c Environmental Science and Technology Research Center, Department of Environmental Health Engineering, School of Health, Shahid Sadoughi University of Medical Sciences, Yazd, Iran

**Keywords:** Humic acid, Heterogeneous catalyst, Photocatalytic, Solar light, Visible light

## Abstract

Nowadays, the presence of humic acid (HA) in water sources is highly regarded due to the production of extremely harmful byproducts such as trihalomethanes. In this study, the effectiveness of an Ag_3_PO_4_/TiO_2_ catalyst produced by in situ precipitation as a heterogeneous catalyst for the degradation of humic acid in the existence of visible and solar light was evaluated. The Ag_3_PO_4_/TiO_2_ catalyst's structure was characterized using X-ray powder diffraction (XRD), scanning electron microscopy (SEM), and energy dispersive spectroscopy (EDS), after which the catalyst dosage, HA concentration, and pH parameters were adjusted. After a 20-min reaction, the highest HA degradation of 88.2% and 85.9% in presence of solar light and visible light were attained at the ideal operating conditions of 0.2 g/L catalyst, 5 mg/L HA, and pH 3, respectively. It was discovered that, based on kinetic models, the degradation of HA matched both Langmuir-Hinshelwood and *pseudo*-first-order kinetics at concentrations of 5 to 30 mg/L (R^2^ > 0.8). The Langmuir-Hinshelwood model had surface reaction rate constants (*K*_*c*_) of 0.729 mg/L.min and adsorption equilibrium constants (*K*_*L-H*_) of 0.036 L/mg. Eventually, a real-water investigation into the process' effectiveness revealed that, under ideal circumstances, the catalyst had a reasonable HA removal efficiency of 56%.

## 1 Introduction

One of the current environmental issues is the existence of organic contaminants in water supplies, including humic acids (HA). One of the primary components of natural organic matter (NOM) is humic acid, a non-uniform macromolecular polymer [1,2]. In both surface and groundwater, HA is a complicated molecule that is frequently present. Humic acid concentrations in drinking water have been measured to be between 2 and 15 mg/L [3,4].

Humic acids include organic functional groups including hydroxyl, carboxyl, carbonyl, methoxy, and quinone groups that can interact with organic and inorganic contaminants in water through mechanisms like absorption, ion exchange, and chelation, resulting in a decline in water quality [[5], [6], [7]]. HA can dissolve in water in very acidic circumstances, whereas fulvic acid is entirely soluble in water [6,8]. As a result, it is crucial to remove HA from water sources since it serves as a precursor to disinfection byproducts, which is a challenging task. In order to remove HA from aquatic environments, several researchers have utilized a variety of techniques, including chemical coagulation [9], electrocoagulation [6], adsorption [[10], [11], [12], [13], [14]], ion exchange [15], membrane processes or membrane separation [[16], [17], [18]], Fenton [19,20], and photocatalysis [3,21,22]. Among these techniques, advanced oxidation processes might be considered.

Advanced oxidation processes generate free radicals with strong oxidizing activity, and as a result, organic compounds are destroyed into simple, low-danger molecules. The complete degradation of organic materials, lack of toxicity, absence of sludge generation, formation of compounds with strong oxidizing capabilities, non-corrosiveness of equipment's pieces, and simplicity of application are all benefits of this process [23,24]. Nanoparticles called nanocatalysts are utilized in advanced oxidation processes to boost effectiveness [25].

Heterogeneous catalysts provide a variety of advantages including the capacity to be reused, high stability, simplicity in separation, and cost-effectiveness. They also help the oxidation process proceed more quickly and efficiently [26,27]. Ultrasound, UV, heat, electricity, and other forms of energy are employed to activate these nanocatalysts.

In recent years, there has been a lot of interest in the photocatalytic destruction of organic molecules and environmental contaminants. Due to its unique qualities in bonding position and surface structure, chemical stability, and lack of toxicity, TiO_2_ is one of the most well-known photocatalysts among other semiconductors. However, due to the high band gap distance and TiO_2_'s inability to use them, visible photons, which make up a considerable portion of the solar spectrum, are limited in their practical uses. In order to develop visible light photocatalysts, a variety of metal oxides, such as Ag_3_PO_4_ with a short band gap spacing, which provides new possibilities for photon gathering in the visible range, can be combined with TiO_2_. As a novel visible light absorption semiconductor, silver orthophosphate (Ag_3_PO_4_), which has a dark golden hue, has frequently garnered attention [[28], [29], [30]].

Although various nanoparticles, including FeNi_3_@SiO_2_@TiO_2_ [31], Mn_2_O_3_–Al_2_O_3_ [32], PAC-LaFeO_3_-Cu [33], Fe_3_O_4_/TiO_2_–N-GO [34], Ce–ZrO_2_ [35], Ag/ZnO [21,36], CuO–Co_3_O_4_@AC [37], FeNi_3_@SiO_2_ [38] have so far been utilized as a catalyst in the degradation of humic acids.

The removal of humic acid using Ag_3_PO_4_/TiO_2_ nanoparticles in the presence of solar light and visible light has not yet been reported. Contrary to earlier research, which employed ultraviolet light radiation for this nanoparticle. In this work, visible light and solar light were utilized to boost the effectiveness of this nanoparticle since they are more accessible, less expensive, and environmentally friendly than other methods. As a result, the goal of this study was to provide a low-cost environment in which to lower the amount of HA in water. Since spectrophotometric measurement is a convenient and effective method for detecting HA, it was also utilized in this study. The effects of the initial HA concentration, the dosage of nanoparticles, and the initial pH value on HA degradation were also investigated.

## 2 Materials and methods

### 2.1 Chemicals and instruments

Merck Company (Germany) supplied silver nitrate (AgNO_3_). Degussa (Germany) provided the P25 titania (TiO_2_), while Sigma-Aldrich provided the Na_3_PO_4_ for the catalyst production. Sigma-Aldrich was also used to purchase the humic acid, sodium hydroxide (NaOH), and hydrogen chloride (HCl). The pH of the solutions was adjusted with HCl and NaOH (1 *N*), and the pH was detected with a pH meter (Wegtech Mi 151 22, UK). At a maximum wavelength of 254 nm, a UV–vis spectrophotometer (Model Optima SP3000 Plus, Japan) was utilized to measure the concentration of HA. The structure of this yellow nanocomposite, which acts as a heterogeneous catalyst, was studied using XRD, SEM, and EDS tools. XRD (Bourevestnik-Dron8) was used to characterize the shape and phase of a nano-crystal catalyst, SEM (TESCAN-Vega3) was used to study the structure, morphology, and surface characteristics of the nano-catalyst on the nanoscale, and EDS (TESCAN-Vega3) was used to evaluate the component mass percentages at the nano-catalyst and BET to quantify the size of a particular area in the nanocatalyst (BELSORP MINI II). After validating the physical and chemical composition of the heterogeneous nanocatalyst, it was utilized to degrade HA in an aqueous media.

### 2.2 Preparation of Ag_3_PO_4_/TiO_2_

The in situ precipitation approach developed by Yao et al. was used to synthesize silver phosphate (Ag_3_PO_4_) deposition onto titania (P25) [39]. In 50 mL of deionized water, 1.6 g of titania (P25 with 80% anatase and a surface area of 50 m^2^/g) was disseminated and sonicated for 5 min. Following sonication, 3.05 g of AgNO_3_ was introduced to the titania disseminated water and magnetically agitated for 10 min at 200 rpm. The sodium phosphate was distributed in 50 mL of deionized water before being dropped into the reaction mixture. For 300 min, the finished solution was magnetically agitated. The color of the mixture was then altered from white to yellow. After that, the Ag_3_PO_4_/TiO_2_ nanocomposite was strained, rinsed with water and ethanol, and dried for 12 h at 60 °C. After preparing the heterogeneous catalyst, it was utilized in a photocatalytic process and characterization.

### 2.3 Batch photocatalytic experiments

The photocatalytic process was influenced by pH, catalyst dose, initial HA concentration, and contact duration, which were all investigated and modified. From a stock HA solution with a 500 mg/L concentration, HA concentrations of 5, 10, and 25 mg/L were produced. To find the optimal catalyst dose, the study examined 0.1, 0.15, 0.2, and 0.3 g/L. The experiments examined pH values of three to eleven (3, 7, and 11), as well as contact times of 3, 5, 10, 20, 30, and 45 min. The experiment was conducted using a 250 mL aluminum-covered container with a 20-W bulb lamp for visible light, then with the same container without the aluminum cover in direct solar light. The following formulas were used to calculate the degrading efficiency of HA (Eq. (1)):

(Eq.1) $$Removal\;Efficiency\;\left(\%\right)=\frac{C_{0}-C_{t}}{C_{0}}\times 100$$

Where the concentrations of HA before and after contact time are shown by *C*_*0*_ and *C*_*t*_ (mg/L) [40].

### 2.4 Determine the pH_zpc_

The pH_zpc_ was determined utilizing 50 mL of KCl 0.1 M mixture at six pHs (2, 4, 6, 8, 10, and 12) and 0.01 g of heterogeneous catalyst nanoparticles. After 24 h, set the prepared mixtures on the shaker and take the pH reading. Utilizing the formula ΔpH = pH_final_-pH_initial_, the resulting curve was displayed as X = initial pH and Y = ΔpH. The pH_zpc_ is the X-axis point where the curve intersects [41].

## 3 Results and discussion

### 3.1 Characterization of Ag_3_PO_4_/TiO_2_

**Fig. 1:**
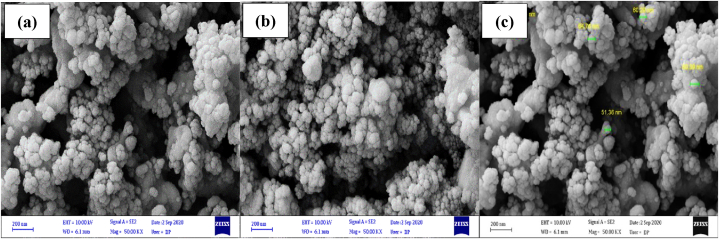
SEM images (a–c) of Ag_3_PO_4_/TiO_2_ heterogeneous catalyst.

The FESEM images of the synthesized Ag_3_PO_4_/TiO_2_ heterogeneous catalyst were used to assess the morphology, size, and shape. FESEM images of the Ag_3_PO_4_/TiO_2_ heterogeneous catalyst synthesized are shown in Fig. 1(a–c). The Ag_3_PO_4_/TiO_2_ heterogeneous catalyst structure was formed into a sphere-shaped and nano-heterogeneous catalyst that was loosely aggregated, uniformly, and smoothly. The particle size of this catalyst is shown in Fig. 1c. It may be stated that the mean particle size of Ag_3_PO_4_/TiO_2_ heterogeneous catalyst is 50–60 nm according to the particle size.

**Fig. 2:**
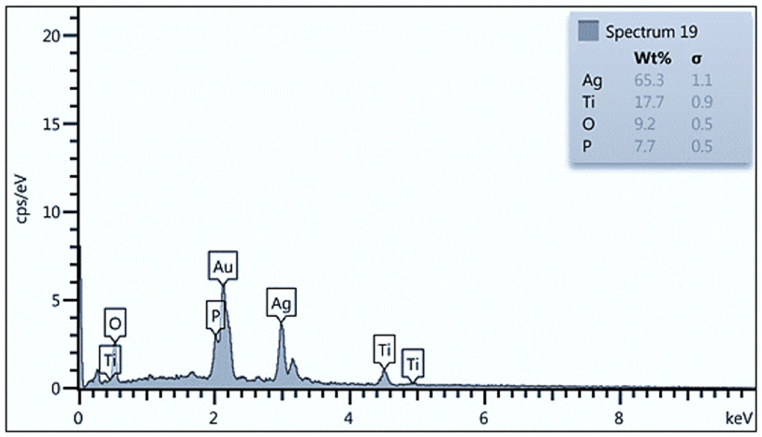
EDS of Ag_3_PO_4_/TiO_2_ heterogeneous catalyst.

EDS analysis was used to assess the homogeneity and chemical structure of the produced Ag_3_PO_4_/TiO_2_ heterogeneous catalyst (Fig. 2). The EDS results show that the chemical structure of Ag_3_PO_4_/TiO_2_ heterogeneous catalyst contains 65.3% Ag, 17.7% Ti, 9.2% O, and 7.7% P, which are all within the predicted range.

**Fig. 3:**
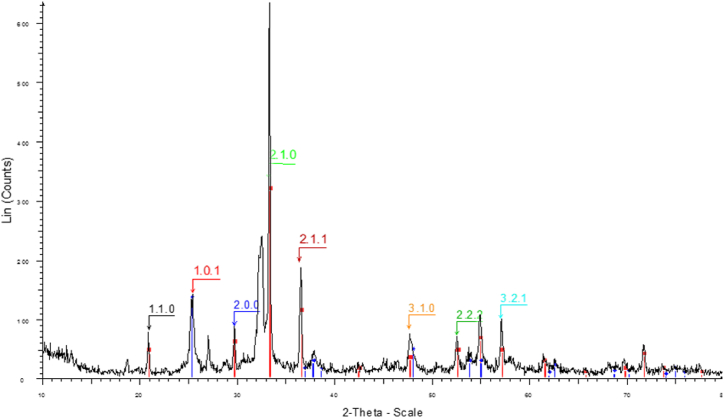
XRD of Ag_3_PO_4_/TiO_2_ heterogeneous catalyst.

XRD analysis was performed to characterize the Ag_3_PO_4_/TiO_2_ heterogeneous catalyst's phases, structures, and crystalline constitution. Fig. 3 depicts the XRD findings. Based on the Joint Committee on Powder Diffraction Standards (JCPDS 96-591-0064), the Ag_3_PO_4_/TiO_2_ heterogeneous catalyst crystal phase structure and XRD pattern with diffraction peaks at 2θ, except for the peak at 25.3° indexed to the (101) plane of TiO_2_ (P25), additional peaks at 2θ values of 20.93° (110), 29.78° (200), 33.39° (210), 36.68° (211), 47.94° (310), 52.85° (222), 55.19° (321) indexed to Ag_3_PO_4_. Furthermore, the observed peak at 2θ∼32.5° is attributable to the lattice distortion in the structure of the nanocatalyst caused during its synthesis. The Ag_3_PO_4_/TiO_2_ heterogeneous catalyst crystal structure was successfully conserved, according to the findings. Its proper and high-performance synthesis was demonstrated by the findings of this nanocatalyst's characterization, which were extremely comparable and consistent to those of the nanocatalyst synthesized by Taheri et al. [24].

**Fig. 4:**
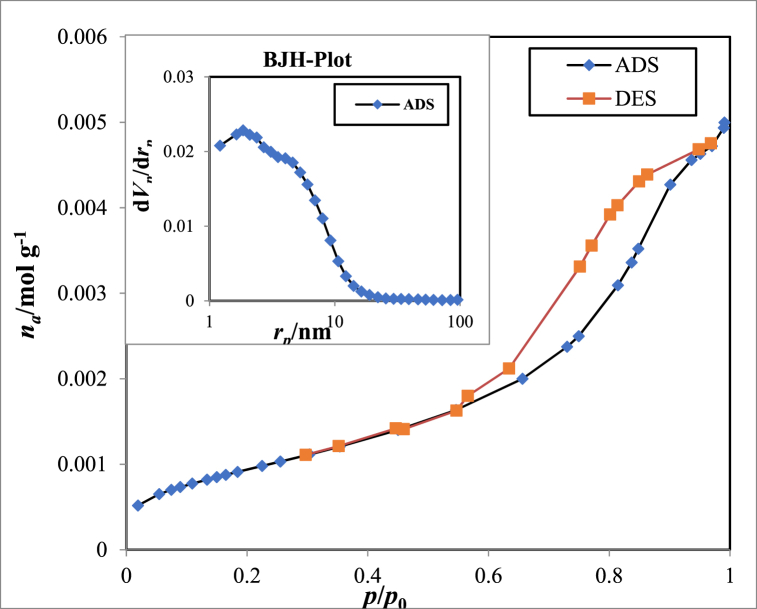
Adsorption/desorption isotherm and BJH surface area of Ag_3_PO_4_/TiO_2_ nanocatalyst.

The Ag_3_PO_4_/TiO_2_ adsorption/desorption isotherm and the BJH specific surface area are shown in Fig. 4. To determine the Ag_3_PO_4_/TiO_2_ nanocatalyst's BET surface area, N_2_ adsorption/desorption studies were conducted. The monolayer volume of gas adsorbed was calculated using the BET equation, which can then be used to determine the surface area of the adsorbent [42]. The total pore volume and BJH of the produced nanocatalyst (*p/p*_*0*_ = 0.990) were calculated using the ADS/DES plot. This value was 47.138 m^2^/g.

### 3.2 Optimization of effective parameters on HA degradation

#### 3.2.1 Effect of catalyst dose

**Fig. 5:**
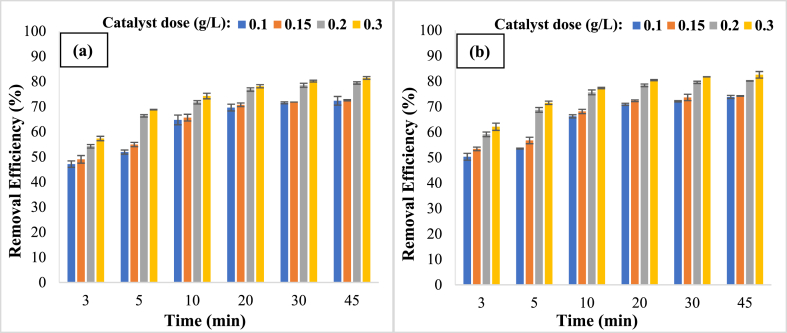
Comparison of removal efficiency at different catalyst doses in presence of visible (a) and solar (b) light (HA concentration: 5 mg/L, pH: 7).

Catalyst dosages of 0.1 to 0.3 g/L were investigated in the study of HA degradation. Fig. 5 shows that increasing the Ag_3_PO_4_/TiO_2_ catalyst dosage from 0.1 to 0.3 g/L enhanced the removal effectiveness of HA from 69.6% to 78.1% for visible light and 70.9% to 80.4% for solar light in 20 min. The formation of additional hydroxyl radicals by the catalyst in the reaction medium may be the explanation for improving the effectiveness of HA degradation by raising the catalyst dosage. On the other hand, by raising the catalyst dosage from 0.2 to 0.3 g/L, the efficiency rises slightly from 76.8% to 78.1% in visible light and 78.4% to 80.4% in solar light, which might be attributed to a limitation of HA leaving in the reaction medium [[43], [44], [45]]. So, the 0.2 g/L of catalyst dose was considered an optimal catalyst dose. The presence of UV rays in sunlight explains how sunlight has a better efficacy of elimination than visible light. This beam excites the majority of electrons on the catalyst's surface and ultimately releases their energy, forcing water molecules to generate hydroxyl (^●^OH) and superoxide (O^●^_2_^-^) radicals, which then break down the HA molecules [46]. The findings of this study accord with those of Nasiri et al. which discovered that when the catalyst dosage is increased, the removal efficiency improves as well [47].

#### 3.2.2 Effect of HA initial concentration

**Fig. 6:**
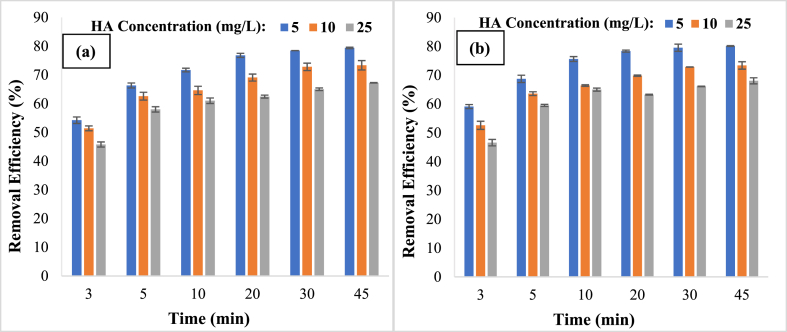
Comparison of removal efficiency at different HA initial concentrations in presence of visible (a) and solar (b) light (Catalyst dose: 0.2 g/L, pH: 7).

Concentration levels of 5, 10, and 25 mg/L were studied for the initial influence of HA on degrading effectiveness. Fig. 5 shows that when the concentration of HA increases from 5 to 25 mg/L, its degradation effectiveness declines from 76.8% to 62.5% for visible light (Fig. 6a) and from 78.4% to 63.3% for solar light (Fig. 6b) in 20 min. This was because raising the concentration of HA increased the formation of intermediates and by-products. However, while the quantity of ^●^OH and O^●^_2_^-^ generated in the reaction medium stayed unchanged, the significant inclination of by-products and intermediates to react with ^●^OH and O^●^_2_^-^ hindered additional decomposition of HA in the reaction medium, lowering its degradation effectiveness [41,48]. As a consequence, 5 mg/L of HA was shown to be the optimal initial concentration. Similar findings were achieved in research conducted by Khodadai et al. on the photocatalytic degradation of HA, and it was discovered that as the concentration of HA increases, its removal effectiveness decreases [49].

#### 3.2.3 Effect of pH on HA degradation

**Fig. 7:**
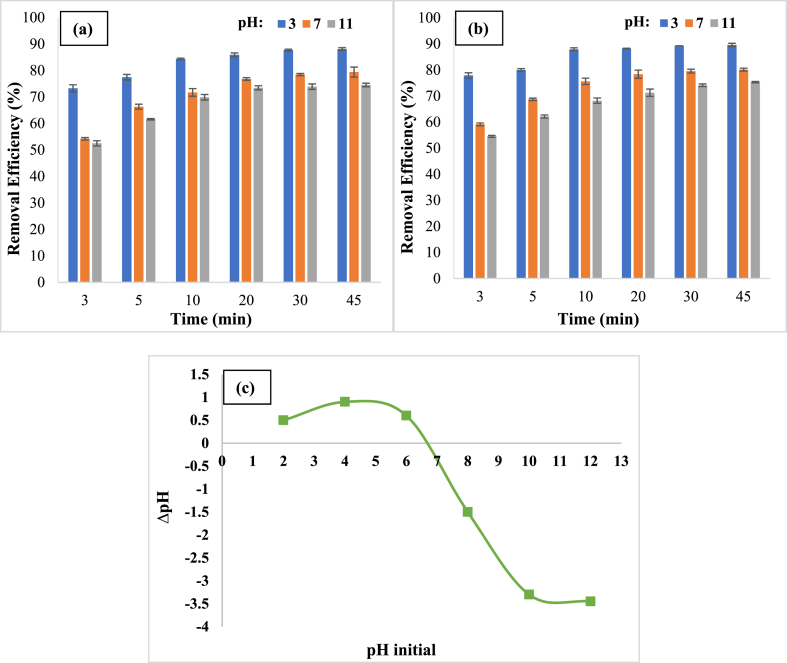
Comparison of removal efficiency at different pHs in presence of visible (a), solar (b) light, and pH_zpc_ (c) (Catalyst dose: 0.2 g/L, HA concentration: 5 mg/L).

pH is a critical parameter that influences the solubility of HA, kinetics, radical generation, and catalyst surface characteristics. pHs 3, 7, and 11 were investigated to see how pH affected the removal effectiveness of HA. In this section, it was discovered that raising the pH from 3 to 11 reduces the removal effectiveness of visible light from 85.6% to 73.5% and solar light from 88.2% to 71.3% in 20 min (Fig. 7a and b). The insolubility and precipitation of HA in the reaction media, which is attributable to the nature of this organic matter, is one of the reasons for improving its removal effectiveness at acidic pH. When HA decomposes in the reaction medium, mineral compounds such as CO_2_ are generated, and CO_2_ is transformed to HCO_3_^−^ in alkaline media, where it consumes active and energetic hydroxyl radicals. It generates CO^●^_3_^-^ radicals since it has decreased oxidation potential, lowering HA elimination efficiency (Eq. (2)) [46]. Furthermore, the surface charge of the catalyst turns negative at pHs over 6.7 and positive at pHs under 6.7, according to the catalyst's pH_zpc_ of 6.7 (Fig. 7c). Humic acid, on the other hand, has two pKa_1_ = 4 and pKa_2_ = 8, and it forms positively charged ions at pHs less than 4, zwitterion or neutral at pHs between 4 and 8, and negatively charged ions at pHs greater than 8, so at an optimum pH of 3, there are repulsive electrostatic forces, and HA is an organic molecule and hydrophobic interactions are dominant mechanism for near HA molecules to the catalyst surface, ultimately leading to degradation [50,51]. Similar results were achieved in Mohtar et al. investigation of the degradation of humic acid, confirming the findings of this study [52].

(Eq. 2) *HCO*_*3*_^*−*^*+*^*●*^*OH → CO*^*●*^_*3*_^*-*^*+ H*_*2*_*O*

### 3.3 Kinetic study of HA degradation

The *pseudo*-first-order kinetic (Eq. (3)) and Langmuir-Hinshelwood (Eq. (4)) models were used to examine HA degradation kinetics. The reactions that take place on the surface of reactants have different adsorption processes as well as rate equations that are essential for heterogeneous catalysis. One of the most frequent models for explaining heterogeneous catalytic processes is the Langmuir-Hinshelwood kinetic. The contaminant adsorption on catalyst active sites is studied in this model [53].

(Eq. 3) $$\ln\frac{C_{t}}{C_{0}}=-K_{obs}t$$

Where *C*_*0*_ and *C*_*t*_ (mg/L) are the initial and after-contact HA concentrations, respectively, and *K*_*obs*_ is the reaction rate constant (min^−1^).

(Eq. 4) $$\frac{1}{K_{obs}}=\frac{1}{K_{C}K_{L-H}}+\frac{C_{0}}{K_{C}}$$

Where *K*_*c*_ represents the surface reaction rate constant (mg/L.min) and *K*_*L-H*_ represents the adsorption equilibrium constant (L/mg). The value of *K*_*obs*_ was calculated for the various concentrations displayed in Table 1 by graphing *ln* (*C*_*t*_*/C*_*0*_) vs time [45].

**Table 1:** *pseudo*-first-order kinetic parameters of HA degradation.

C_0_ (mg/L)	R^2^	K_obs_	Line Equation
5	0.837	52.356	y = −0.0191× - 1.6445
10	0.890	64.516	y = −0.0155× - 1.746
25	0.814	81.301	y = −0.0123× - 1.4367

**Fig. 8:**
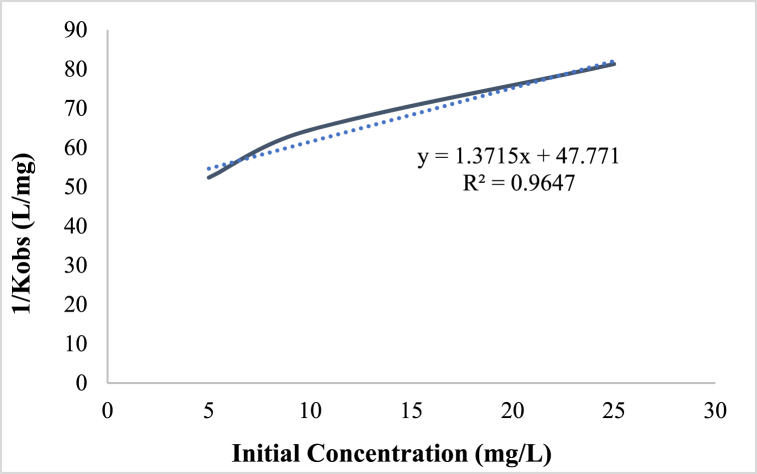
Langmuir-Hinshelwood kinetic curve (Catalyst dose: 0.2 g/L, HA concentration: 5 mg/L, pH: 3, and time: 20 min).

Then, utilizing the linear equation derived by graphing the curve *K*_*obs*_^*−1*^ vs the initial concentration depicted in Fig. 8, the coefficients of *K*_*c*_ and *K*_*L-H*_ were computed. The coefficients of *K*_*c*_ and *K*_*L-H*_ were 0.729 mg/L.min and 0.036 L/mg, respectively, based on the result acquired from this curve. These findings also revealed that HA degrades according to *pseudo*-first-order and Langmuir-Hinshelwood kinetics. Research on humic acid decomposition was performed by Kamani et al. Humic acid decomposition follows pseudo-first-order and Langmuir-Hinshelwood kinetics, according to the study [54].

### 3.4 Investigation of process efficiency on real water

The impact of the Ag_3_PO_4_/TiO_2_ catalyst process on HA degradation in real water was studied in this research. A sample of real water was obtained from a water treatment plant in Shiraz, Iran, according to parameters NO_3_: 29.5 mg/L, total hardness: 462 mg/L, SO_4_: 164 mg/L, Chloride: 81 mg/L, pH: 7.8, total dissolved solids (TDS): 665 mg/L, and Na: 54 mg/L. The removal effectiveness of HA from real water was 56% and 59.5% in presence of visible and solar light respectively, at the optimum conditions (pH 3, HA concentration 5 mg/L, and catalyst dosage 0.2 g/L) derived from the synthetic sample experiment, indicating the acceptable efficiency of this process in the treatment of real water. The reduced removal effectiveness of HA in real water may be attributed to the existence of impurities including cations, anions, TDS, as well as other impurities, which are removed as a consequence of the oxidation process; on the other hand, there could be inhibiting cations and anions in real water that serve as scavengers and diminish the activity of free radicals [55]. The findings of Acero et al. investigation of real water samples were similar to the findings of this study [56].

### 3.5 Degradation mechanism and stability of nanocatalyst

The heterogeneous catalyst structure between Ag_3_PO_4_ and TiO_2_ can explain the increased photocatalytic activity of Ag_3_PO_4_/TiO_2_ relative to ordinary TiO_2_. The photocatalytic efficiency of TiO_2_ is improved by coating Ag_3_PO_4_ on its surface, which enhances light absorption and enables electron-hole segregation according to their corresponding band orientations [57,58]. The border potentials of Ag_3_PO_4_'s conduction band (CB) and valence band (VB) are +0.45 and + 2.9 eV, respectively, which are higher than TiO_2_ (−0.5 and + 2.7 eV, respectively) [[59], [60], [61]]. Either Ag_3_PO_4_ and TiO_2_ can be activated when exposed to light. Photon-generated holes in Ag_3_PO_4_'s VB rapidly move to TiO_2_, while photon-generated electrons move to Ag_3_PO_4_'s CB. So, to the imposed electric field, photon-generated holes in the VB of Ag_3_PO_4_ might move to TiO_2_ during light irradiation, whilst Ag_3_PO_4_ serves as a sensitizer absorbing light [45,[62], [63], [64]]. As a result, the electron-hole coupling mechanism is hindered, resulting in increased photoactivity of Ag_3_PO_4_/TiO_2_. Under solar and visible light irradiation, the proposed mechanism of photocatalytic decomposition of HA over Ag_3_PO_4_/TiO_2_. Photon-generated holes in Ag_3_PO_4_ particles easily move to the VB of TiO_2_ when exposed to light. Since the VB value of TiO_2_ (+2.7 eV) is more positive than the typical redox potential E° (OH/OH^−^, 1.99 eV), the holes in the VB of TiO_2_ may oxidize OH^−^ or H_2_O to produce OH radicals (as demonstrated in Eqs. (Eq. 3), (Eq. 4) [51,65]. Nevertheless, the CB of Ag_3_PO_4_ (+0.45 eV) is lower than the normal redox potential of E° (O_2_/H_2_O_2_) (0.682 eV), implying that the electrons in the CB of Ag_3_PO_4_ can be transported to O_2_ molecules adsorbed on the photocatalysts' surfaces and produce H_2_O_2_ [66]. As seen in Eqs. (Eq. 5), (Eq. 6), H_2_O_2_ combines with electrons in a series to form active ^●^OH to some amount. As a result, strongly oxidative species such as ^●^OH and holes are generated, which react with the HA in aqueous media (Eq. (7)) [43,67].

*h*^*+*^*+ H*_*2*_*O →*^●^*OH + H*^*+*^ (Eq. 3)

*h*^*+*^*+ OH*^*−*^*→*^●^*OH* (Eq. 4)

(Eq. 5) *2e*^*-*^*+ O*_*2*_*+ 2H*^*+*^*→ H*_*2*_*O*_*2*_

(Eq. 6) *e*^*-*^*+ H*_*2*_*O*_*2*_*→*^*●*^*OH + OH*^*−*^

(Eq. 7) ^*●*^*OH, h*^*+*^*+ HA → CO*_*2*_*+ H*_*2*_O + …

**Fig. 9:**
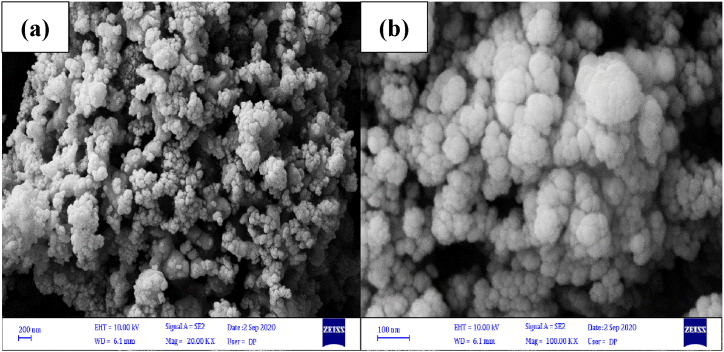
SEM images after finishing process (a and b).

The nanocatalyst was filtered from the reaction media once the experiment was finished, and its structure was analyzed using SEM analysis (Fig. 9 a and b). According to the analysis's findings, the stability of the nanocatalysts was shown by the fact that their morphology was conserved once the reaction was over. The concentration of Ag (328.1 nm) and Ti (363.4 nm) were determined using an Atomic Absorption Spectrophotometer (AAS, CTA-3000) following the reaction in an aqueous solution to ascertain the chemical stability of Ag_3_PO_4_/TiO_2_. The measurements made using this device's findings show that the concentration of Ag was 4.5 mg/L and Ti was below the AAS detection limit, indicating that this nanocatalyst has the necessary chemical stability [68,69].

To assess the main radicals in the humic acid degradation process, the radical scavenger's benzoquinone (BQ) and *tert*-butanol (TBA) were utilized for superoxide (^•^O_2_^−^) and hydroxyl radicals (^•^OH), respectively [70]. The elimination efficiency dropped from 88.2% to 61.5% when BQ was added to the reaction medium in the presence of solar light, and it dropped to 31.6% when TBA was added. This indicates that hydroxyl radicals were the dominant radicals in the degradation of humic acid.

### 3.6 Comparison with other catalysts to HA degradation

As indicated in Table 2, due to the experimental settings, the procedure examined in this study has a higher removal efficiency than in previous studies. Solar light has been utilized as a catalytic activator in all of the processes. Based on the findings of previous research, the amount of catalyst used in this study was reasonable given the high removal effectiveness and short contact time, and it was also higher cost-effective than some others.

**Table 2:** Comparison of the performance process VS other processes in HA degradation.

No.	Catalyst	Catalyst dose (g/L)	Initial Concentration (mg/L)	Time (min)	Removal Efficiency (%)	Ref.
1	NiCo_2_O_4_	0.2	10	120	90	[71]
2	PAC/LaFeO_3_/Cu	0.72	5.33	36.2	95.5	[72]
3	PE-TiO_2_	6.0	10	270	64.5	[73]
4	CeO_2_/AC	0.5	50	30	75	[74]
5	TiO_2_/Fe_2_O_3_	0.4	20	180	61.6	[75]
6	CuO–Co_3_O_4_@AC	0.5	100	60	88	[76]
7	Ag/ZnO	0.6	50	40	70	[77]
8	Ag_3_PO_4_/TiO_2_	0.2	5	20	88.2	This study

## 4 Conclusion

A simple and effective approach was used to produce Ag_3_PO_4_/TiO_2_ as a nano-heterogeneous catalyst. SEM, XRD, and EDS analyses were used to characterize the structure of this nano-heterogeneous catalyst. The particle size was 50–60 nm on the mean, according to SEM. The crystal structure of this catalyst was effectively retained, according to XRD measurements. Under optimum conditions, this catalyst removed humic acid from the aqueous medium with an efficiency of 88.2% and 85.9% in presence of solar light and visible light, respectively: catalyst dosage 0.2 g/L, starting concentration 5 mg/L, and pH 3. The decomposition of HA follows all these kinetics with coefficients of determination (R^2^) of 0.837, 0.890, and 0.814 at concentrations of 5, 10, and 25 mg/L, respectively, according to the *pseudo*-first-order kinetic and Langmuir-Hinshelwood model. *K*_*c*_ and *K*_*L-H*_ were also discovered to be 0.729 mg/L.min and 0.036 L mg, respectively. This catalyst's and process's effectiveness indicated that it may be used to remove other organic compounds.

## Author contribution statement

Roya Morovati and Saeed Rajabi: Conceived and designed the experiments; performed the experiments; analyzed and interpreted the data; contributed reagents, materials, analysis tools or data; wrote the paper.

Mohammad Taghi Ghaneian and Mansooreh Dehghani: Conceived and designed the experiments; analyzed and interpreted the data; contributed reagents, materials, analysis tools or data; wrote the paper.

## Data availability statement

Data will be made available on request.

## Declaration of interest’s statement

The authors declare no conflict of interest.
